# Weekly osimertinib dosing prevents *EGFR* mutant tumor cells destined to home mouse lungs

**DOI:** 10.1016/j.tranon.2021.101111

**Published:** 2021-05-13

**Authors:** Ashwin Butle, Asim Joshi, Vanita Noronha, Kumar Prabhash, Amit Dutt

**Affiliations:** a Integrated Cancer Genomics Laboratory, Advanced Centre for Treatment Research Education In Cancer (ACTREC), Tata Memorial Centre, Navi Mumbai, Maharashtra, India 410210; b Department of Medical Oncology, Tata Memorial Centre, Ernest Borges Marg, Parel, Mumbai, India 400012; c Homi Bhabha National Institute, Training School Complex, Anushakti Nagar, Mumbai, India 400094

**Keywords:** ADAURA trial, Adjuvant EGFR-TKI treatment, Orthotopic mouse lung cancer model, Tail vein injection, Bioluminescence imaging

## Abstract

The recently conducted ADAURA trial concludes daily dosing of adjuvant osimertinib, a third-generation EGFR tyrosine kinase inhibitor (TKI), improves disease-free survival with stage IB/II/IIIA *EGFR* -mutated non-small cell lung cancer patients in comparison to placebo. We have developed a preclinical orthotopic mouse model, using luciferase tagged lung adenocarcinoma cells harboring EGFR TKI sensitive exon 19 deletion to model and extend trial implications comparing a weekly vs daily dosing outcome of osimertinib to a first-generation TKI- erlotinib. We find that 100% of mice in both the groups receiving osimertinib daily or weekly before injection of cells show a complete absence of homing of cells in mice's lungs from day three until day 18 post-injection of cells. On the other hand, 25% and 75% of mice receiving erlotinib daily and weekly before injecting cells show homing of cells to the lungs. The tumors observed in the lungs, when dissected at day 30, confirmed the colonization of the injected cells homing to the organ. Thus, our study establishes the efficacy of pretreatment with osimertinib in reducing tumor cells' homing to mouse lungs in an *in vivo* mouse model.

## Highlights

• Human lung cancer cells injected through mouse-tail vein colonize mouse lungs within 24 h and form lung tumors in about 30 days.
• Pretreatment of mice with weekly or daily dosing of erlotinib *reduces but does not abolish* tail vein injected lung cancer cells to form lung tumors.
• Pretreatment of mice with weekly or daily dosing of osimertinib *abolish* lung cancer cells injected through a tail vein to form lung tumors.
• The low-dose once-a-week adjuvant osimertinib could have lower toxicity, higher affordability, and potentially affect acquired resistance.

## Graphical abstract

**Figure:**
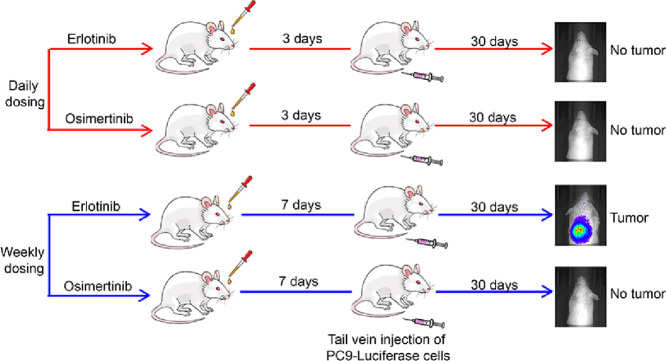

The ADAURA trial reveals that daily dosing of osimertinib as adjuvant treatment in previously untreated *EGFR* mutation-positive NSCLC patients, with stage IB to IIIA, extends disease-free survival compared to patients in the placebo group [1]. We set to establish an appropriate preclinical orthotopic mouse model using luciferase tagged lung adenocarcinoma PC9 cells, harboring EGFR TKI sensitive exon 19 deletion, injected through a tail vein to model and extend the implications of the ADAURA trial. The extent of reduction in the cells that would otherwise home to the lungs of animals within 24 h post-injection allowed us to compare the benefit of osimertinib, a third-generation epidermal growth factor receptor tyrosine kinase inhibitor (TKI), to the first-generation TKI erlotinib along with their daily vs weekly dosing efficacy.

Tail vein mice models present an attractive tool for studying lung cancer pathogenesis and has been employed to develop orthotopic lung tumors and study lung metastasis [2]. Using an experimental *in vivo* mice model system, we present an assessment of the homing of *EGFR* mutant lung cancer cells to the lungs of the mice injected intravenously through the tail vein. Thirty NOD-SCID mice, six to eight weeks old, with bodyweight in the range of 18 g to 22 g were divided into five groups of six mice each. Two groups were utilized for daily pretreatment dosing regimen, two groups for weekly pretreatment dosing regimen and one group was kept for vehicle control. A comparative account of the relevant pharmacokinetic parameters (Supplementary Table S1 and S2) suggest osimertinib, unlike erlotinib, can be detected in plasma and brain of male rats even after 21 days of a single oral dose of 5 mg/kg [3]. Thus, for all the mice in the respective groups, erlotinib (25 mg/kg) and osimertinib (15 mg/kg) were orally administered, as described earlier [4,5], as a single or weekly dose at days indicated in Fig. 1. Following the dosing regimens of EGFR-TKIs, at day 0, 2 × 10^6^ PC9-luciferase cells were injected into the tail vein of mice. Although six mice were taken per group, one mouse from control and two from each treatment group died during the tail vein injections, most likely due to thromboembolism, as reported earlier [6]. Thus, five mice in the control group and four mice in each treatment group were used for further experiments. In the control group, 100% of the mice showed homing and retention of cells in the lungs of mice 18 days post-injection of PC9-luciferase cells. The absence of luminescence signal in the middle days and its re-emergence in a couple of mice in the control group could be because of the lower number of cells in the lungs below the detection limit of IVIS imaging system. The tumors were observed in the lungs when dissected at day 30, confirming the colonization of the injected cells homing to the organ.

**Fig. 1:**
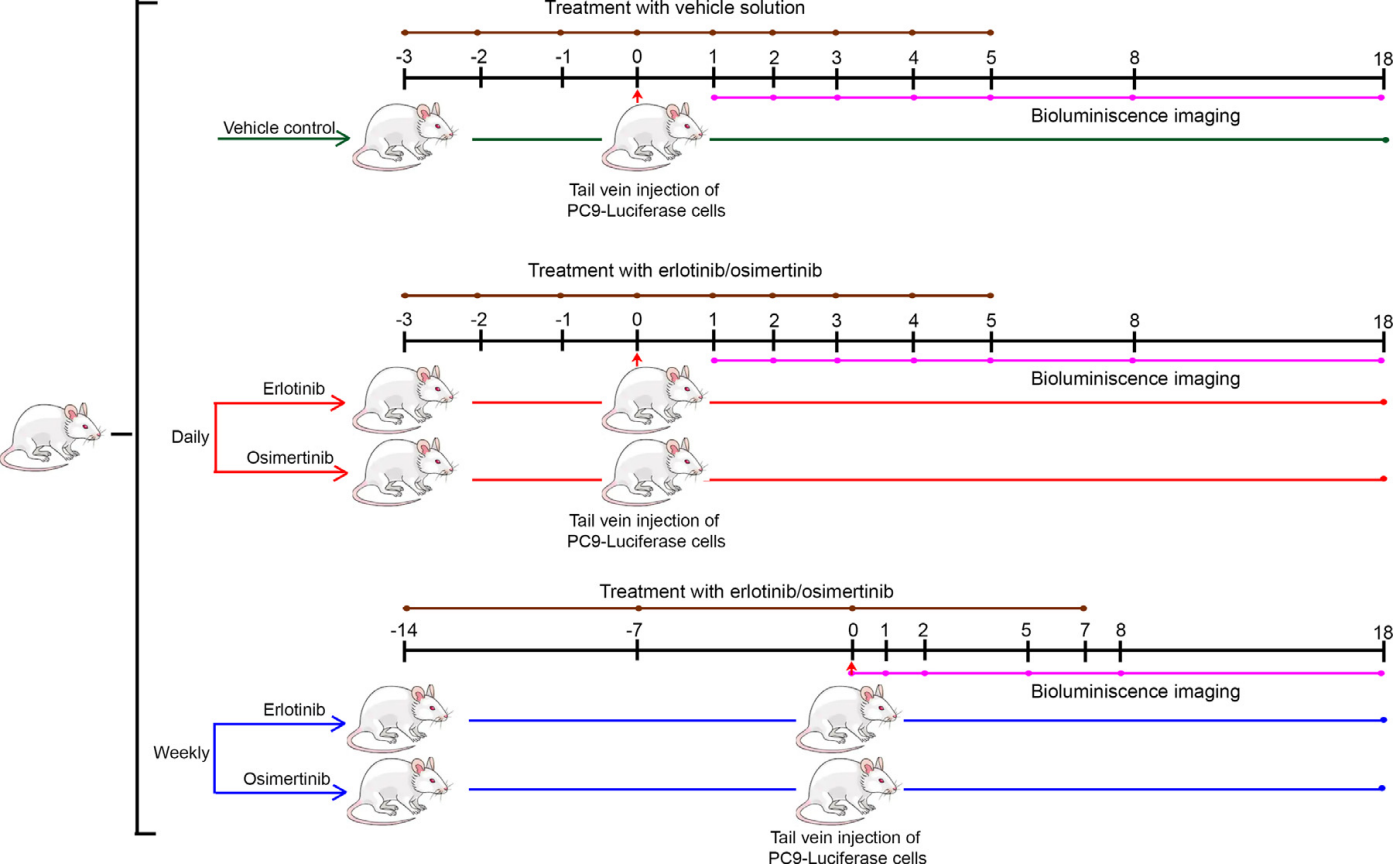
Schematic overview of experimental strategy to assess the utility of EGFR-TKIs in preventive settings. NOD-SCID mice were divided into three groups (*n* = 6 in each group) according to different dosing regimens of daily (red) and weekly (blue) doses of EGFR-TKIs. A vehicle control group is indicated in green. For daily dosing, mice received EGFR-TKIs daily three days before injecting PC9 cells (indicated by red arrow). The treatment was continued daily till day 5 post-injection. For the weekly dosing regimen, EGFR-TKIs treatment was given once per week for two weeks before injecting PC9 cells and continued for one-week post-injection. The bioluminescence imaging was performed on respective days as indicated by the dots on pink line for different therapeutic regimens. For daily dosing and vehicle control group, bioluminescence imaging was performed on days 0, 1, 2, 3, 4, 5, 8 and 18 post PC9 injection. Weekly bioluminescence imaging was performed on day 0, 1, 2, 5, 8 and 18 days post PC9 injection.

In the case of mice treated with erlotinib weekly, only one mouse showed an absence of homing of cells in the lungs of mice. In three of four mice, the homing of cells in the mice was observed even after day 18 post-injection. The median time to recurrence among NSCLC patients is 25 months after stopping erlotinib [7]. However, daily pretreatment with erlotinib could significantly prevent the homing of cells as only a single mouse of four among the erlotinib daily pretreatment group showed recurrence of observable PC9 cells in the lungs of mice (Fig. 2). On day 18 post-injection in one mouse, we observed the bioluminescence signal's re-emergence, possibly indicating the emergence of the resistant cells. This could also be attributed to residual PC9 cells' growth after discontinuation of erlotinib treatment on day 5. The outcome is reminiscent of the SELECT, a phase II trial of adjuvant erlotinib in *EGFR* mutant NSCLC patients (*N* = 100), wherein the disease recurred in 4 of 40 patients. Of note, the bioavailability of erlotinib has been shown to vary significantly in individuals, and because of its low bioavailability, erlotinib is used at very high doses to many excipients [8,9]. Moreover, high dose weekly erlotinib, in the range of 1000 mg – 1500 mg, effectively controls CNS metastases in *EGFR* mutant lung cancer patients [10,11]. The ineffectiveness of weekly erlotinib dosing in our study is possibly because of same dose of erlotinib used in daily and weekly treatment regimens. Chemotherapeutic agents, on the other hand, show a plural response with increased efficacy as reported for a once-a-week dose of paclitaxel in breast and ovarian cancer [12,13] or once every-3-weeks cisplatin in head and neck cancer [14], but not for docetaxel in breast and prostate cancer [15].

**Fig. 2:**
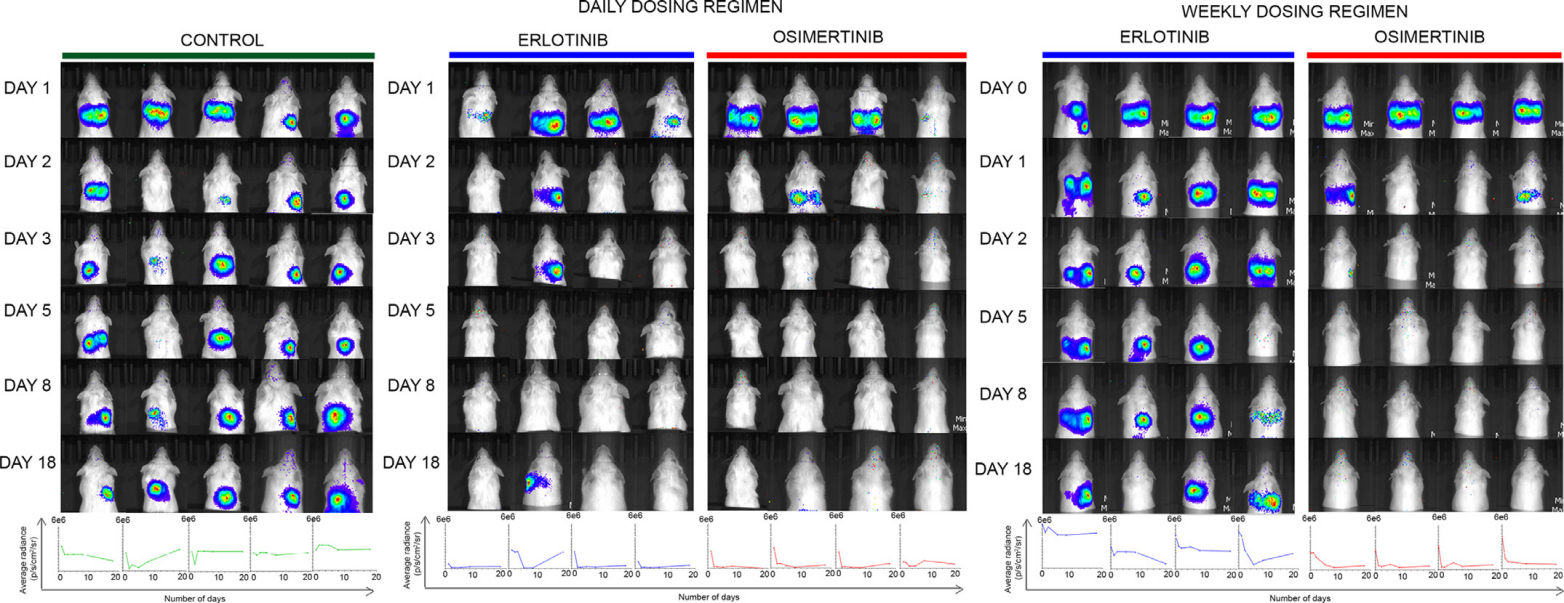
Bioluminescence images of NOD-SCID mice intravenously injected with 2 × 10^6^ PC9 cells after pretreatment with TKI inhibitors. Successful tail vein injections were observed in five mice in the control group and four mice in the erlotinib and osimertinib group. Bioluminescence images were taken after injecting 100 ul of 30 mg/ml luciferin intraperitoneally into the mice. Images for each mice taken on day 1, day 3, day 5, day 8 and day 18 post-injection of cells are shown. The images are taken after covering the tail to eliminate the background luminescence emerging from cells blocked in the tail of mice. The graph below each mice images represents time in days plotted against average radiance (p/s/cm^2^/sr) for each mice. The y-axis scale is split into three segments, segment 1 from 0 to 20,200, segment 2 from 1e^5^ to 5e^5^ and segment 3 from 2e^6^ to 6e^6^. The x-axis indicates the number of days from 0 to 20.

Interestingly, 100% of the mice in both the groups receiving osimertinib daily and weekly showed a complete absence of homing of cells in the lungs of mice from day 3 post-injection of cells. Unlike the erlotinib group, in both the osimertinib groups, none of the mice showed re-appearance of bioluminescence signal till day 18 post injections suggesting osimertinib as more effective in reducing the homing of cells in the mice. In the weekly osimertinib pretreatment group, only a single oral dose after cell injection showed absence of recurrence and disease-free survival of mice as observed till day 18 with no luminescence observed even on day 30 (data not shown). With these encouraging observations following osimertinib pretreatment, we believe that this study may help rationalize and improve our understanding of the ADAURA trial. The efficient clearance of PC9 cells from the lungs with osimertinib may be due to delayed metabolism, maximum bioavailability in tumor generation site, and the lungs. Osimertinib has also been reported to undergo minimal first-pass metabolism with low clearance and high distribution volume in humans, possibly accounting for the efficacy of osimertinib in the weekly dosing group [16].

Overall, based on the complete absence of homing of cells in lungs of osimertinib pre-treated mice, in both weekly and daily regimens, EGFR-TKI pretreatment may represent a viable treatment option in delaying the onset of disease in patients as an adjuvant treatment post resection of early-stage tumors or among patients with pre-disposition to *EGFR* mutant lung cancers harboring germline *EGFR* kinase domain mutations [17,18]. Besides, the low-dose once-a-week osimertinib could potentially have several advantages over daily dosing, including lower toxicity, affordability, ease of administration and delaying or preventing acquired resistance that remains to be explored.

## Funding

This work was supported by an extramural grant from SERB-DST (EMR/2016/007218) to A.D. The funders had no role in study design, data collection and analysis, decision to publish and preparation of the manuscript.

## Author contributions

A.B, A.J and A.D. designed the study, A.B. and A.J. performed experiments, A.B, A.J, V.N, K.P. and A.D. analyzed and interpreted the data. A.B, A.J. and A.D. wrote the manuscript. A.D. further certifies that for this study A.B. and A.J. should be considered first-authors to all academic and professional effects.

## Ethics approval

All experimental procedures performed in this study followed ethical guidelines for animal studies and were approved by the Institutional Animal Ethical Committee of ACTREC (Proposal No. 9/2020).

## Declaration of Competing Interest

None declared

## Appendix Supplementary materials

Image, application 1

## References

[1] Wu Y.L., Tsuboi M., He J., John T., Grohe C., Majem M. (2020). Osimertinib in Resected EGFR-Mutated Non-Small-Cell Lung Cancer. N. Engl. J. Med..

[2] Mendoza A., Gharpure R., Dennis J., Webster J.D., Smedley J., Khanna C. (2013). A novel noninvasive method for evaluating experimental lung metastasis in mice. Journal of the American Association for Laboratory Animal Science: JAALAS.

[3] Ballard P., Yates J.W., Yang Z., Kim D.W., Yang J.C., Cantarini M. (2016). Preclinical Comparison of Osimertinib with Other EGFR-TKIs in EGFR-Mutant NSCLC Brain Metastases Models, and Early Evidence of Clinical Brain Metastases Activity. Clin. Cancer Res..

[4] Gu J., Yao W., Shi P., Zhang G., Owonikoko T.K., Ramalingam S.S. (2020). MEK or ERK inhibition effectively abrogates emergence of acquired osimertinib resistance in the treatment of epidermal growth factor receptor-mutant lung cancers. Cancer.

[5] Tan J., Li M., Zhong W., Hu C., Gu Q., Xie Y. (2017). Tyrosine kinase inhibitors show different anti-brain metastases efficacy in NSCLC: A direct comparative analysis of icotinib, gefitinib, and erlotinib in a nude mouse model. Oncotarget.

[6] Shrestha N., Lateef Z., Martey O., Bland A.R., Nimick M., Rosengren R. (2019). Does the mouse tail vein injection method provide a good model of lung cancer?. F1000Research..

[7] Pennell N.A., Neal J.W., Chaft J.E., Azzoli C.G., Janne P.A., Govindan R. (2019). SELECT: A Phase II Trial of Adjuvant Erlotinib in Patients With Resected Epidermal Growth Factor Receptor-Mutant Non-Small-Cell Lung Cancer. J. Clin. Oncol..

[8] Yang K.M., Shin I.C., Park J.W., Kim K.S., Kim D.K., Park K. (2017). Nanoparticulation improves bioavailability of Erlotinib. Drug Dev Ind Pharm.

[9] Wu Q., Li M.Y., Li H.Q., Deng C.H., Li L., Zhou T.Y. (2013). Pharmacokinetic-pharmacodynamic modeling of the anticancer effect of erlotinib in a human non-small cell lung cancer xenograft mouse model. Acta Pharmacol. Sin..

[10] Grommes C., Oxnard G.R., Kris M.G., Miller V.A., Pao W., Holodny A.I. (2011). Pulsatile" high-dose weekly erlotinib for CNS metastases from EGFR mutant non-small cell lung cancer. Neuro-oncology.

[11] Clarke J.L., Pao W., Wu N., Miller V.A., Lassman A.B. (2010). High dose weekly erlotinib achieves therapeutic concentrations in CSF and is effective in leptomeningeal metastases from epidermal growth factor receptor mutant lung cancer. J. Neurooncol..

[12] Katsumata N., Yasuda M., Takahashi F., Isonishi S., Jobo T., Aoki D. (2009). Dose-dense paclitaxel once a week in combination with carboplatin every 3 weeks for advanced ovarian cancer: a phase 3, open-label, randomised controlled trial. Lancet.

[13] Sparano J.A., Wang M., Martino S., Jones V., Perez E.A., Saphner T. (2008). Weekly paclitaxel in the adjuvant treatment of breast cancer. N. Engl. J. Med..

[14] Noronha V., Joshi A., Patil V.M., Agarwal J., Ghosh-Laskar S., Budrukkar A. (2018). Once-a-Week Versus Once-Every-3-Weeks Cisplatin Chemoradiation for Locally Advanced Head and Neck Cancer: A Phase III Randomized Noninferiority Trial. J. Clin. Oncol..

[15] Rivera E., Mejia J.A., Arun B.K., Adinin R.B., Walters R.S., Brewster A. (2008). Phase 3 study comparing the use of docetaxel on an every-3-week versus weekly schedule in the treatment of metastatic breast cancer. Cancer.

[16] Vishwanathan K., So K., Thomas K., Bramley A., English S., Collier J. (2019). Absolute Bioavailability of Osimertinib in Healthy Adults. Clin Pharmacol Drug Dev.

[17] de Alencar V.T.L., Formiga M.N., de Lima V.C.C. (2020). Inherited lung cancer: a review. Ecancermedicalscience.

[18] Gazdar A., Robinson L., Oliver D., Xing C., Travis W.D., Soh J. (2014). Hereditary lung cancer syndrome targets never smokers with germline EGFR gene T790M mutations. J. Thorac. Oncol..

