# Supplementary material for: Weekly osimertinib dosing prevents EGFR mutant tumor cells destined to home mouse lungs
Source: Transl Oncol. 2021 May 13;14(8):101111. doi: 10.1016/j.tranon.2021.101111 (PMC8236545; doi:10.1016/j.tranon.2021.101111)
Supplement: Supplementary file 1 [file mmc1.docx]

**Weekly Osimertinib Dosing Prevents *EGFR* Mutant Tumor Cells Destined to Home Mouse Lungs**

**Supplementary Materials and Methods**

**Cell culture and Inhibitors**

PC-9 lung adenocarcinoma cells were cultured in RPMI medium (Gibco, Catalog no. 23400-021) supplemented with 10% fetal bovine serum (Gibco, Catalog No. 10270-106), 2.5mg/ml Amphotericin-B (Abbott) and 1.5ul/ml gentamycin (Abbott) and incubated at 37 ^O^C in a 5% CO_2_ incubator. The identity of the cells was confirmed by DNA short tandem repeat profiling kit (Promega, Geneprint 10 System). The chromosomal mutation and drug sensitivity status of the PC9 cells were re-confirmed by directed sequencing of *EGFR* exon 19 and by performing MTT assay, shown in supplementary figure S1, as described earlier (1) OAD2007_5': CTTGTGGAGCCTCTTACACCC and OAD2008_3': ATCTGCACACACCAGTTGAG primers were used to amplify the amplicon. The cells were tested for mycoplasma and, whenever necessary were treated as per the EZKill mycoplasma elimination kit (Himedia, Catalog no. CCK006). PC-9 cells were tagged with luciferase by infection with lentiviral particles produced using the vector pLenti CMV Puro Luc EGFR tyrosine kinase inhibitors, erlotinib (Santacruz biotechnology) and osimertinib (Selleckchem, Catalog No. S7297), dissolved in 10% DMSO in 1X PBS for animal experiments.

***In vivo* studies and bioluminescence imaging**

The animal experiments and study protocols were reviewed and approved by the Institutional Animal Ethics Committee of ACTREC, Tata Memorial Center. All the animals were housed in the laboratory animal facility at ACTREC, Tata Memorial Center. Female NOD-SCID mice (6-8 weeks old) were grouped into three groups of six mice, i) Control group, ii) Erlotinib group and iii) Osimertinib group. The mice in the respective groups were treated with vehicle control (10% DMSO in 1X PBS), Erlotinib (25mg/kg of body weight) and Osimertinib (15 mg/kg of body weight). The vehicle control and inhibitor treatments were administered orally using a gavage needle. Different pretreatment modalities with inhibitors, including a) daily pretreatment for three days and b) weekly treatment was given two weeks before injecting the cells. Following the respective pretreatment modalities, all the mice were anaesthetized using isoflurane inhalation and intravenously injected with 2 X 106 luciferase tagged PC9 cells suspended in 80ul PBS using a 30G hypodermic syringe. In case of daily pretreatment, the erlotinib/osimertinib treatment was continued for five days post-injection of cells, after which the treatment was stopped. For the weekly pretreatment modality, the treatment was continued till one-week post-injection of cells. The homing of the cells in the lungs of the mice was observed using bioluminescence imaging (Caliper LifeSciences, IVIS Spectrum). For bioluminescence imaging, mice were intraperitoneally injected with 100 μl of 30 mg/ml of luciferin (carbosynth, L-8220) and anaesthetized using isoflurane inhalation. Imaging was performed using the auto exposure mode in (Living Image 4.5) until the peak was reached. The mice were imaged every day till five days post-injection, after which the imaging was performed on day 8 and day 18 for the daily pretreatment group while, in case of the weekly pretreatment group, mice were imaged for day 0, 1, 2, 5, 8 and 18. To negate the luminescence emitted from the cells blocked in the tail and capture the luminescence emitting from the lungs only, the tail of the mice was covered with black paper while imaging.

**Supplementary Figure S1**


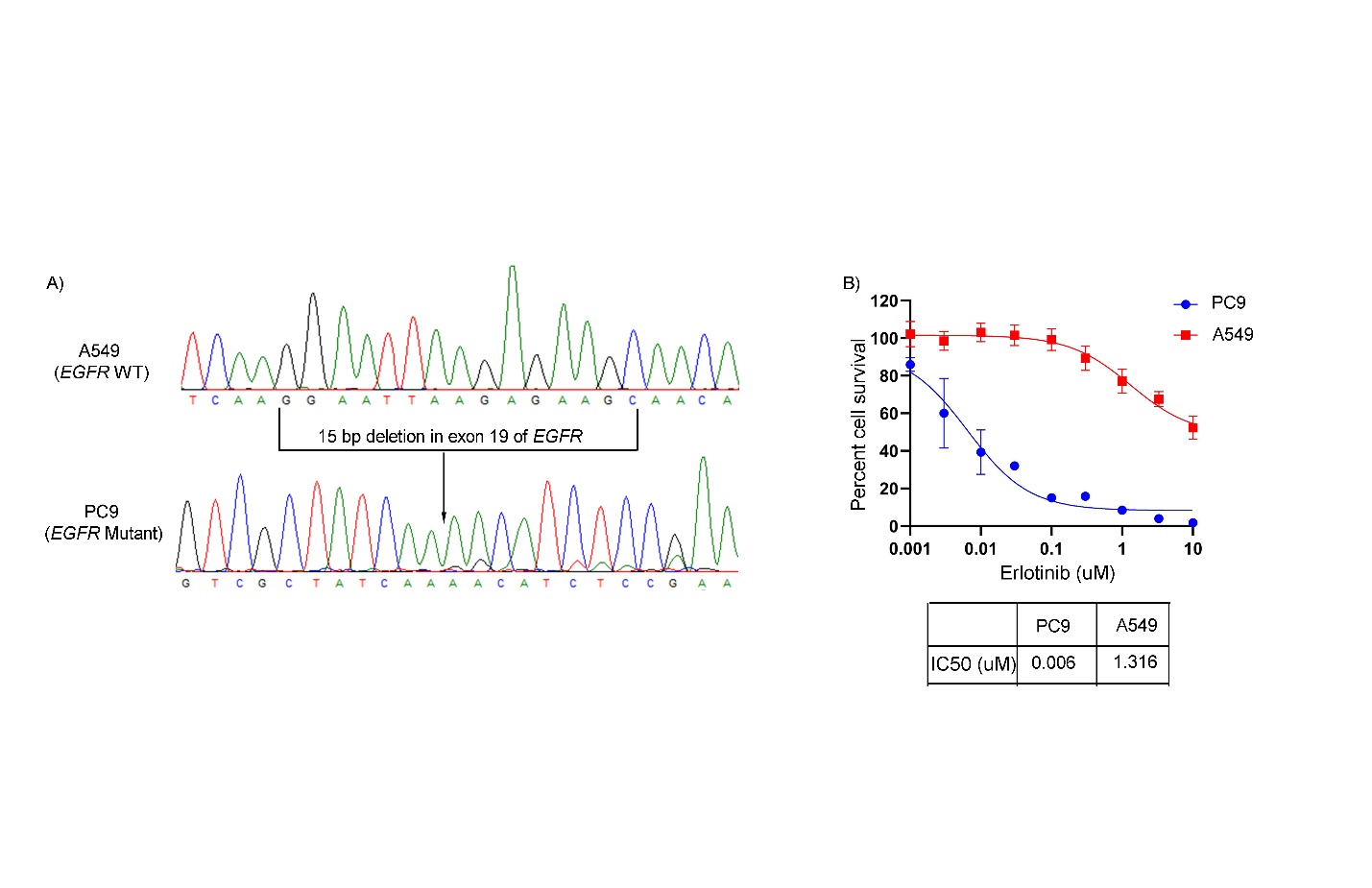
**Supplementary Figure S1: Confirmation of chromosomal mutation status and drug sensitivity status of PC9 cells.** A) The 15bp deletion in the exon 19 of *EGFR* in PC9 cells was confirmed by Sanger sequencing. The sequenced bases from PC9 cells were compared with the identical region of A549 cells, harboring wild type *EGFR.* B) MTT assay was used to confirm the sensitivity of PC9 cells to erlotinib. A549 cells were used as an erlotinib resistant control.

**Supplementary Tables**

**Supplementary Table S1:** Pharmacokinetic values of erlotinib and osimertinib in animal models

| **TKIs** | **Half-Life** | **Animals** | **Dose** | **Dosing regimen** | **Bioavailability in Plasma** | | | **Ref.** |
| --- | --- | --- | --- | --- | --- | --- | --- | --- |
|  |  |  |  |  | **Concentration**  **( C_max_)** | **Time**  **( T_max_)** | **Clearance**  **(Cl)** |  |
| **Erlotinib** | 36 h | Female, BALB/c | Oral, (10 mg kg) | Single | 2323 ng/ml | 0.5 h | NA | (2) |
|  |  | Female BALB/c nude | Oral, (12.5 mg kg) | Single | NA | NA | 0.739 L·kg^-1^·h^-1^ | (3) |
|  |  | Male beagle dogs | Oral,(100mg/10kg) | Single | 2385 ± 493 ng/ml | 1.25 ± 0.50 h | NA | (4) |
|  |  | SCID mice | I.P. (30 mg kg) | Single | 49994.17±2928.14 ng/ml | 0.33 h | 102.10±3.52 ml/h | (5) |
|  |  | Female SCID mice | Gefitinib  Oral,(6.25 mg kg) | Daily, 8weeks | Plasma 0.82,  Brain 0.17 µM/L | NA | NA | (6) |
|  |  | Male rats | Oral, (5 mg kg) | Single | 0.36 nmol eq/g at 6h, Brain:blood ratio: 0.69 at 6h & was below lower limit of quantification at 24h | | | (6) |
| **Osimertinib** | 48 h | Female SCID mice | Oral, (5 mg kg) | Daily, 8weeks | Plasma: 1.92 µM/L | 0.5 h | NA | (6) |
|  |  |  |  |  | Brain: 1.03 ng/ml | 2 h | NA |  |
|  |  |  |  |  | Tumor: 0.69 ng/ml | 4 h | NA |  |
|  |  |  | Oral, (25 mg kg) | Daily, 8weeks | Plasma: 2.98 ng/ml | 0.5 h | NA |  |
|  |  |  |  |  | Brain: 7.13 ng/ml | 4 h | NA |  |
|  |  |  |  |  | Tumor: 5.79 ng/ml | 4 h | NA |  |
|  |  | Male rats | Oral, (4 mg kg) | Single | Brain:blood ratio= (2.2 at 60 min, 0.2 up to 21 Days, below the lower limit of quantification at 60 Days) | | | (6) |
|  |  | Female Partially Pigmented Rats | Oral, (4 mg kg,  Radio-labelled) | Single | Radioactivity (blood)= 0.5h: 1.00, 1h: 1.00, 6h: 1.00, 24h: 1.00, 2d: 1.00, 7d: 1.00, 21d: 1.00  Radioactivity (lungs)= 0.5h: 14.0, 1h: 21.3,6h: 20.4, 24h: 3.11, 2d: 1.24, 7d: 0.85, 21d: 0.81 | | | (7) |
|  |  | Male Albino Rats |  |  | Radioactivity (blood)= 1h: 1.00 ,6h: 1.00, 24h:1.00  Radioactivity (lungs)=,1h: 25.2 ,6h: 33.9, 24h: 3.21 | | |  |
|  |  | Female Albino Rats |  |  | Radioactivity (blood)= 1h: 1.00, 6h: 1.00, 24h: 1.00  Radioactivity (lungs)=,1h: 37.3, 6h: 43.1, 24h: 6.10 | | |  |
|  |  | Healthy male volunteers | Oral, (20 mg, Radio-labelled) | Single | 29.9 nM | 6.00 h | 26.7 L/h | (7) |
|  |  | Male Sprague-Dawley rats | Oral, (10 mg kg) | Single | 51.47 ± 10.74 µg/L | 4.60±2.41 h | 19.76 ±5.84 L/h/Kg | (8) |
|  |  | Male Sprague-Dawley rats | Oral, (10 mg kg) | Single | 0.317 ± 0.138 μg/mL | 3.33 ± 0.82 h | 19.821 ± 8.132 L/h/kg | (9) |
|  |  | Male Sprague-Dawley rats | Oral, (4.5 mg kg) | Single | 21.89 ± 6.05 ng/mL | 3.23 ± 0.77 h | 33.72 ± 16.13  L/h/kg | (10) |

**Supplementary Table S2:** Effective concentration of erlotinib and osimertinib in rodent models for therapeutic approach

| **TKIs** | **Animal** | **Dose (mg/kg)** | **Dosing regimen** | **Time ( Days)** | **Ref.** |
| --- | --- | --- | --- | --- | --- |
| **Erlotinib** | Female BALB/c nude | Oral, (50 mg kg) | Daily | 30 | (3) |
|  | female BALB/c nude mice | Oral , (10mg in water) | Daily | 60 | (4) |
|  | Female BALB/c nu/nu nude mice | Oral, (50 mg kg) | Daily | 20-50 | (11) |
|  | C57BL/6J mice | I.P. (30 mg/kg) | Daily | 21 | (12) |
|  | Transgenic mice*, TetO-EGFR^L858R^* | I.P. (25 mg/kg) | 5 days/week | 28 | (13) |
|  | Transgenic mouse, Myf6Cre | Oral, (100 mg kg) | Daily | 2-3weeks | (14) |
|  | SOD1 transgenic mouse | I.P. (75 mg/kg) | Daily | 9 weeks | (15) |
|  | Nude female mice | Oral (5 & 50 mg/kg) | Daily | 15 | (16) |
|  | Female SPF BALB/c nude mice | Oral, (**25** mg kg) | Daily | 4 weeks | (17) |
|  | BALB/c nu/nu mice | Oral, (25 mg kg) | Daily | NA | (18) |
|  | Nude female mice | Oral, (12.5 mg kg) | Daily | 6 | (19) |
|  | Male Sprague-Dawley rats | Oral, (20 mg kg) | Daily | 2 weeks | (20) |
| **Osimertinib** | Athymic nu/nu nude mice | Oral, (25 mg kg) | Daily | 14 | (21) |
|  | Female SCID mice | Oral, (5 & 25 mg kg) | Daily | 56 | (6) |
|  | Athymic nu/nu nude mice | Oral, (25 mg kg) | Daily | 28 | (22) |
|  | Nude female mice | Oral, (5 mg/kg) | Daily | 15 | (16) |
|  | Male mice | Oral, (25 mg kg) | Every 4^th^ day | NA | (23) |
|  | Balb/c-Nude female mice | Oral, (3 mg kg) | 5 days/week | NA | (24) |
|  | BALB/c nude mice | Oral, (2.5 & 5 mg kg) | Twice/week | 16 | (25) |
|  | Nude mice | Oral, (15 mg kg) | Daily | 83 | (26) |

**References**

1. Iyer P, Shrikhande SV, Ranjan M, Joshi A, Gardi N, Prasad R, et al. ERBB2 and KRAS alterations mediate response to EGFR inhibitors in early-stage gallbladder cancer. International journal of cancer. 2019;144(8):2008-19.

2. Smith NF, Baker SD, Gonzalez FJ, Harris JW, Figg WD, Sparreboom A. Modulation of erlotinib pharmacokinetics in mice by a novel cytochrome P450 3A4 inhibitor, BAS 100. Br J Cancer. 2008;98(10):1630-2.

3. Wu Q, Li MY, Li HQ, Deng CH, Li L, Zhou TY, et al. Pharmacokinetic-pharmacodynamic modeling of the anticancer effect of erlotinib in a human non-small cell lung cancer xenograft mouse model. Acta Pharmacol Sin. 2013;34(11):1427-36.

4. Yang KM, Shin IC, Park JW, Kim KS, Kim DK, Park K, et al. Nanoparticulation improves bioavailability of Erlotinib. Drug development and industrial pharmacy. 2017;43(9):1557-65.

5. Patel SD, Patel UD, Sadariya KA, Bhavsar SK, Thaker AM. Impact of Erlotinib and Metformin Administration on Pharmacokinetics of Meloxicam in SCID Mice. International Journal of Current Microbiology and Applied Sciences. 2018;7(03):2252-60.

6. Ballard P, Yates JW, Yang Z, Kim DW, Yang JC, Cantarini M, et al. Preclinical Comparison of Osimertinib with Other EGFR-TKIs in EGFR-Mutant NSCLC Brain Metastases Models, and Early Evidence of Clinical Brain Metastases Activity. Clinical cancer research : an official journal of the American Association for Cancer Research. 2016;22(20):5130-40.

7. Dickinson PA, Cantarini MV, Collier J, Frewer P, Martin S, Pickup K, et al. Metabolic Disposition of Osimertinib in Rats, Dogs, and Humans: Insights into a Drug Designed to Bind Covalently to a Cysteine Residue of Epidermal Growth Factor Receptor. Drug metabolism and disposition: the biological fate of chemicals. 2016;44(8):1201-12.

8. Wu Q, Jiang H, Wang S, Dai D, Chen F, Meng D, et al. Effects of avitinib on the pharmacokinetics of osimertinib in vitro and in vivo in rats. Thoracic Cancer. 2020;11(10):2775-81.

9. Ying Z, Wei J, Liu R, Zhao F, Yu Y, Tian X. An UPLC-MS/MS Method for Determination of Osimertinib in Rat Plasma: Application to Investigating the Effect of Ginsenoside Rg3 on the Pharmacokinetics of Osimertinib. International journal of analytical chemistry. 2020;2020:8814214.

10. Xiong S, Deng Z, Sun P, Mu Y, Xue M. Development and Validation of a Rapid and Sensitive LC-MS/MS Method for the Pharmacokinetic Study of Osimertinib in Rats. Journal of AOAC International. 2017;100(6):1771-5.

11. Friess T, Scheuer W, Hausmann M. Erlotinib antitumor activity in non-small cell lung cancer models is independent of HER1 and HER2 overexpression. Anticancer research. 2006;26(5A):3505-12.

12. Zhang X, Chen J, Jin H, Zhao W, Chang Z, Wu H. Effect of erlotinib combined with cisplatin on IL-6 and IL-12 in mice with Lewis lung cancer. Oncology letters. 2020;20(1):902-6.

13. Ayeni D, Miller B, Kuhlmann A, Ho PC, Robles-Oteiza C, Gaefele M, et al. Tumor regression mediated by oncogene withdrawal or erlotinib stimulates infiltration of inflammatory immune cells in EGFR mutant lung tumors. Journal for immunotherapy of cancer. 2019;7(1):172.

14. Abraham J, Nelon LD, Kubicek CB, Kilcoyne A, Hampton ST, Zarzabal LA, et al. Preclinical testing of erlotinib in a transgenic alveolar rhabdomyosarcoma mouse model. Sarcoma. 2011;2011:130484.

15. Le Pichon CE, Dominguez SL, Solanoy H, Ngu H, Lewin-Koh N, Chen M, et al. EGFR inhibitor erlotinib delays disease progression but does not extend survival in the SOD1 mouse model of ALS. PloS one. 2013;8(4):e62342.

16. Katsuya Y, Miyake K, Higuchi T, Oshiro H, Sugisawa N, Singh SR, et al. Comparison of the Efficacy of EGFR Tyrosine Kinase Inhibitors Erlotinib and Low-dose Osimertinib on a PC-9-GFP EGFR Mutant Non-small-cell Lung Cancer Growing in the Brain of Nude Mice. In vivo. 2020;34(3):1027-30.

17. Tan J, Li M, Zhong W, Hu C, Gu Q, Xie Y. Tyrosine kinase inhibitors show different anti-brain metastases efficacy in NSCLC: A direct comparative analysis of icotinib, gefitinib, and erlotinib in a nude mouse model. Oncotarget. 2017;8(58):98771-81.

18. Tsubata Y, Hayashi M, Tanino R, Aikawa H, Ohuchi M, Tamura K, et al. Evaluation of the heterogeneous tissue distribution of erlotinib in lung cancer using matrix-assisted laser desorption ionization mass spectrometry imaging. Scientific reports. 2017;7(1):12622.

19. Momcilovic M, Bailey ST, Lee JT, Fishbein MC, Magyar C, Braas D, et al. Targeted Inhibition of EGFR and Glutaminase Induces Metabolic Crisis in EGFR Mutant Lung Cancer. Cell reports. 2017;18(3):601-10.

20. Yamamoto Y, Iyoda M, Tachibana S, Matsumoto K, Wada Y, Suzuki T, et al. Erlotinib attenuates the progression of chronic kidney disease in rats with remnant kidney. Nephrology, dialysis, transplantation : official publication of the European Dialysis and Transplant Association - European Renal Association. 2018;33(4):598-606.

21. Higuchi T, Oshiro H, Zhang Z, Miyake K, Sugisawa N, Katsuya Y, et al. Osimertinib Regresses an EGFR-Mutant Cisplatinum- Resistant Lung Adenocarcinoma Growing in the Brain in Nude Mice. Translational oncology. 2019;12(4):640-5.

22. Higuchi T, Sugisawa N, Park JH, Sun Y, Zhu G, Yamamoto N, et al. Osimertinib regressed an EGFR-mutant lung-adenocarcinoma bone-metastasis mouse model and increased long-term survival. Translational oncology. 2020;13(10):100826.

23. MacLeod AK, Lin, Huang JT, McLaughlin LA, Henderson CJ, Wolf CR. Identification of Novel Pathways of Osimertinib Disposition and Potential Implications for the Outcome of Lung Cancer Therapy. Clinical cancer research : an official journal of the American Association for Cancer Research. 2018;24(9):2138-47.

24. La Monica S, Minari R, Cretella D, Flammini L, Fumarola C, Bonelli M, et al. Third-generation EGFR inhibitor osimertinib combined with pemetrexed or cisplatin exerts long-lasting anti-tumor effect in EGFR-mutated pre-clinical models of NSCLC. Journal of experimental & clinical cancer research : CR. 2019;38(1):222.

25. Liu Y, Xiong Z-C, Sun X, Sun L, Zhang S-L, Ma J-T, et al. Impact of apatinib in combination with osimertinib on EGFR T790M-positive lung adenocarcinoma. Translational Cancer Research. 2019;8(5):2151-63.

26. Gu J, Yao W, Shi P, Zhang G, Owonikoko TK, Ramalingam SS, et al. MEK or ERK inhibition effectively abrogates emergence of acquired osimertinib resistance in the treatment of epidermal growth factor receptor mutant lung cancers. Cancer. 2020;126(16):3788-99.
